# Natural Products Targeting the Androgen Receptor Signaling Pathway: Therapeutic Potential and Mechanisms

**DOI:** 10.3390/cimb47090780

**Published:** 2025-09-19

**Authors:** Sitong Wu, Esveidy Isabel Oceguera Nava, Dennis Ashong, Guanglin Chen, Qiao-Hong Chen

**Affiliations:** Department of Chemistry & Biochemistry, California State University, Fresno, CA 93740, USA

**Keywords:** natural product, androgen receptor, testosterone, antagonism, degradation

## Abstract

The androgen receptor (AR) signaling pathway is the primary driver of prostate cancer initiation and progression, including the development of castration-resistant prostate cancer (CRPC). Because current AR-targeted therapies inevitably encounter drug resistance, novel strategies to suppress AR signaling are urgently needed. Natural products represent a rich and structurally diverse source of bioactive compounds capable of targeting AR at multiple regulatory levels. This review overviews the interactions between natural products and the AR signaling axis through distinct mechanisms, including inhibition of testosterone production and 5α-reductase activity, direct antagonism of AR, and induction of AR degradation. In addition, several compounds disrupt AR nuclear translocation, downregulate AR splice variants, or suppress AR signaling indirectly through epigenetic regulation, microRNA modulation, or interference with co-regulator networks. Preclinical studies provide compelling evidence that these agents can effectively interrupt AR signaling, thereby suppressing prostate cancer growth. However, challenges remain, particularly the limited pharmacokinetic characterization, lack of in vivo validation, and scarcity of clinical studies. Future research should focus on improving bioavailability, exploring synergistic combinations with existing therapies, and advancing well-designed in vivo and clinical investigations. Collectively, these efforts may establish natural products as lead compounds to modulate AR signaling for prostate cancer prevention and treatment.

## 1. Androgen Receptor Signaling Pathway: A Primary Driver of Prostate Cancer

**Figure 1:**
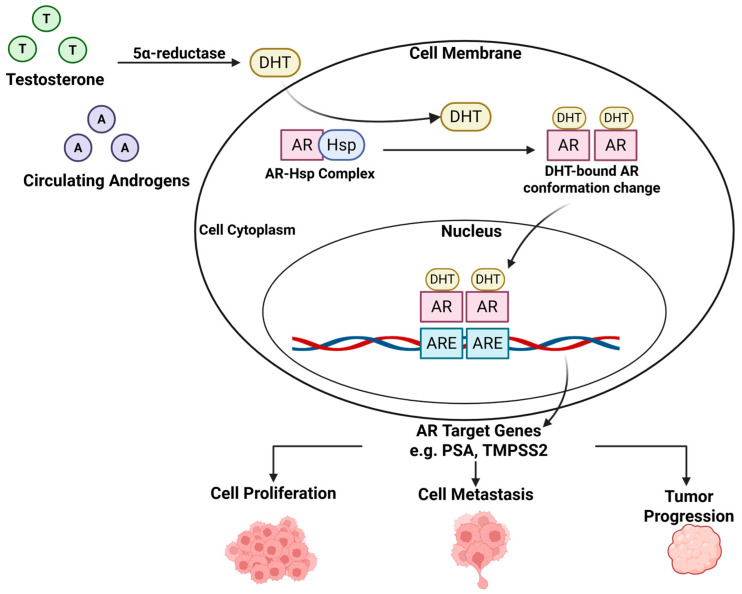
AR signaling pathway and its role in prostate cancer [1]. Created in BioRender. Oceguera nava, E. (2025) https://BioRender.com/6php4lz (accessed on 20 August 2025).

**Figure 2:**
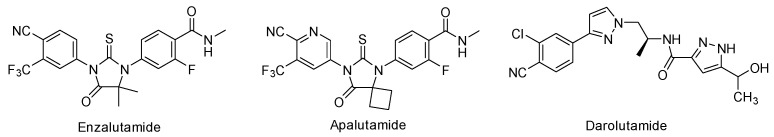
Chemical structures of enzalutamide, apalutamide, and darolutamide.

The androgen receptor (AR) signaling pathway plays a pivotal role in the development and progression of prostate cancer, including advanced forms such as castration-resistance prostate cancer (CRPC) [1]. As illustrated in Figure 1, this pathway is initiated by dihydrotestosterone (DHT), a potent endogenous androgen generated by the 5*α*-reductase-catalyzed reduction in testosterone. Once synthesized, DHT enters prostate cells and binds to AR in the cytoplasm. This ligand binding induces a conformational change in the AR, leading to its dissociation from heat shock proteins (HSP, chaperones), dimerization, and nuclear translocation with the assistance of coactivators containing the LXXLL motif. Inside the nucleus, the DHT-activated AR binds to androgen response elements (AREs), a 15 base pair DNA sequence located in the promoter regions of AR-regulated genes such as prostate specific antigen (PSA) and transmembrane protease serine 2 (TMPRSS2). This ARE binding facilitates the recruitment of specific co-regulators and the assembly of transcription complexes, initiating the transcription of genes involved in prostate cancer cell proliferation, cell metastasis, and tumor progression. Owing to its central involvement in prostate cancer development and progression, the AR signaling pathway is a critical therapeutic target. Drug discovery efforts targeting prostate cancer have focused on androgen deprivation therapy, androgen biosynthesis inhibitors, AR antagonists, AR degraders, and agents that disrupt *N*-terminal and *C*-terminal interactions [2]. Androgen deprivation therapy has been a mainstay in prostate cancer management since its introduction in 1941, typically providing an 18- to 24-month initial response [3]. More recently, the development of three FDA-approved second-generation AR antagonists, enzalutamide [4], apalutamide [5], and darolutamide [6] (Figure 2), has marked significant progress in the treatment of various stages of prostate cancer.

## 2. Natural Products as Potential Treatments for Prostate Cancer

Several naturally occurring compounds, such as genistein, (-)-epigallocatechin-3-gallate (EGCG), curcumin, lycopene, and vitamin D, have been reported to prevent or delay the onset of prostate cancer, as well as development to progression to CRPC [7]. Diverse classes of natural products, including flavonoids, alkaloids, terpenoids, and other polyphenolic compounds, have demonstrated significant antiproliferative activity in prostate cancer cell models in vitro. In some instances, these findings have been supported by the in vivo efficacy in animal models [8]. Growing interest in the mechanistic underpinnings of these effects has led to a couple of comprehensive reviews. Ren and colleagues highlighted the potential of natural AR antagonists in reducing the risk of prostate cancer [9] while Fontana and colleagues summarized the molecular targets and mechanisms by which natural products may exert preventive and therapeutic effects in prostate cancer [10]. The present article focuses specifically on the interactions between natural products and the AR signaling pathway.

## 3. Natural Products Inhibiting Testosterone Production

### 3.1. Biosynthesis of Testosterone and DHT

**Figure 3:**
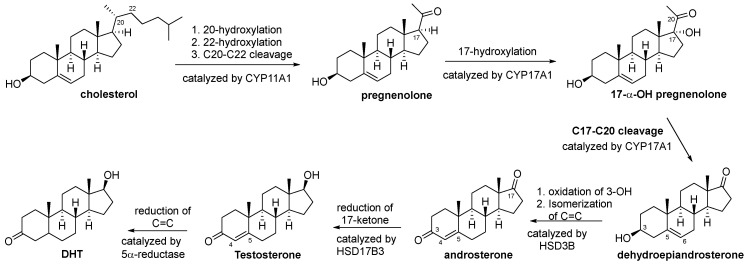
Biosynthesis of testosterone and DHT. CYP11A1 is an enzyme complex consisting of 20-hydroxylase, 22-hydroxylase, and 20-22-lyase. CYP17A1 is an enzyme complex consisting of 17α-hydroxylase and 17,20-lyase. HSD3B is an enzyme complex comprising 3*β*-hydroxysteroid dehydrogenase and ∆^4,5^-isomerase.

As illustrated in Figure 3, testosterone is primarily synthesized by Leydig cells in the testicles from cholesterol through a five-step transformation catalyzed by four different steroidogenic enzymes, including CYP11A1, CYP17A1, HSD3B, and HSD17B3 [11]. Inhibition of any of these enzymes can lead to suppression of testosterone production. Dihydrotestosterone is synthesized by the ∆^4,5^ reduction in testosterone through a process catalyzed by 5α-reductase.

### 3.2. Natural Products Inhibiting the Biosynthesis of Testosterone

**Figure 4:**
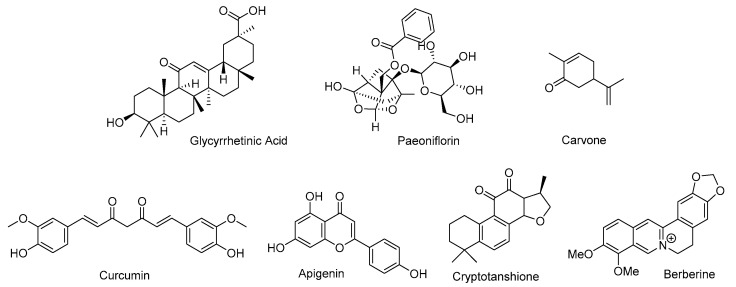
Chemical structures of glycyrrhetinic acid, paeoniflorin, carvone, curcumin, cryptotanshinone, and berberine.

A small number of naturally occurring compounds, glycyrrhetinic acid, paeoniflorin, carvone, curcumin, apigenin, and berberine, have been reported to suppress testosterone biosynthesis through diverse mechanisms (Figure 4). These compounds exhibit distinct chemical structures and originate from various plant sources, yet they converge on similar pathways that modulate androgen production and signaling.

Glycyrrhetinic acid (GA), also known as enoxolone, is a pentacyclic triterpenoid derived from the hydrolysis of glycyrrhizic acid, the active component of licorice (*Glycyrrhiza glabra*). GA acts as a potent inhibitor of key enzymes in the androgen biosynthesis pathway, particularly 17,20-lyase and 17-hydroxysteroid dehydrogenase. This inhibition prevents the conversion of 17-hydroxyprogesterone to dehydroepiandrosterone and its subsequent transformation into testosterone. In vivo studies in castrated male rats treated with *G. glabra* extract (75–300 mg/kg) revealed a dose-dependent reduction in serum testosterone levels [12]. GA also inhibited luteinizing hormone-induced testosterone synthesis in Leydig cells by reducing cAMP levels and suppressing 17-hydroxysteroid dehydrogenase activity [13].

Paeoniflorin, a major bioactive compound found in Chinese white peony, has been shown to enhance aromatase activity, promoting the conversion of testosterone to estrogen. Treatment with paeoniflorin reduced the testosterone/androstenedione ratio in rat ovarian tissue, indicating conversion suppression from androstenedione to testosterone, while estradiol/testosterone levels remained stable [14]. These dual effects, suppression of testosterone synthesis and promotion of estrogen production, was further supported by microsomal assays [13].

Carvone, a monoterpene abundant in spearmint (*Mentha spicata*), has also demonstrated to lower testosterone levels. Traditionally consumed as herbal tea, spearmint reduced plasma testosterone levels in male Wistar rats in a dose-dependent manner, with the highest dose (40 g/L) reducing testosterone from 3.07 ng/mL (control) to 0.73 ng/mL [15]. Clinical studies have echoed these results: in women with hirsutism, five days of spearmint tea consumption lowered free testosterone from 5.49 ± 2.94 pg/mL to 3.92 ± 2.80 pg/mL [15]. In a letrozole-induced PCOS rat model, spearmint oil containing 57.02% carvone significantly decreased serum testosterone, with the lowest levels observed in the group co-treated with 300 mg/kg spearmint oil and letrozole [16].

Curcumin has been shown to suppress androgen biosynthesis and AR signaling through multiple mechanisms. In prostate cancer cells, curcumin downregulates steroidogenic enzymes such as StAR, CYP11A1, and HSD3B2, which are involved in androgen production [17]. Concurrently, curcumin upregulates androgen-inactivating enzymes such as aldo-keto reductase 1C2 (AKR1C2), which catalyzes the conversion of DHT to its inactive form. In LNCaP and 22Rv1 prostate cancer cells, curcumin (10–50 µM) significantly increased AKR1C2 expression, leading to decreased testosterone and DHT levels [17]. These effects were corroborated in vivo using the TRAMP mouse model, where oral administration of curcumin increased prostatic AKR1C2 expression and significantly reduced intratumoral testosterone concentrations.

Apigenin, a dietary flavone abundant in celery and chamomile, also exhibits potent antiandrogenic effects. It inhibits multiple steroidogenic enzymes, including HSD3B, CYP17A1, and HSD17B3, in rat immature Leydig cells [18]. Apigenin significantly suppressed the biosynthesis of 5*α*-androstane-3*α*,17*β*-diol (DIOL), a key androgen metabolite. It demonstrated potent inhibition of HSD3B (IC_50_ values: rat, 11.41 μM; human, 2.17 μM) and HSD17B3 (IC_50_ values: rat, 9.37 μM; human, 1.31 μM), as well as suppression of CYP17A1 activity in both rat and human testicular tissue [18].

Cryptotanshinone, a bioactive compound derived from *Salvia miltiorrhiza*, was revealed to suppress testosterone production by modulating the ERK/c-Fos/CYP17 signaling cascade. This involved the restoration of ERK1/2 and c-Fos expression, downregulation of CYP17, and reductions in androgen levels [19].

Berberine directly inhibits androgen biosynthesis by targeting aldo-keto reductase 1C3 (AKR1C3), a key enzyme in testosterone production also known as 17β-hydroxysteroid dehydrogenase type 5 (17β-HSD5). It significantly suppresses AKR1C3 enzymatic activity (IC_50_ = 4.08 µM), thereby reducing testosterone synthesis and inhibiting proliferation in AKR1C3-overexpressing 22Rv1 cells [20]. Importantly, AKR1C3 is not only central to steroidogenesis but also acts as an androgen receptor (AR)–selective coactivator that is markedly upregulated in castration-resistant prostate cancer (CRPC). In this context, AKR1C3 sustains intratumoral androgen synthesis and reactivates AR signaling despite systemic androgen deprivation, which contributes to enzalutamide resistance [21,22]. Inhibition of AKR1C3 has been shown to re-sensitize resistant prostate cancer cells to enzalutamide and suppress tumor growth [22]. Consistent with these findings, berberine’s suppression of AKR1C3-driven proliferation highlights its potential as a therapeutic strategy to disrupt intratumoral steroidogenesis in CRPC.

## 4. Natural Products Inhibiting 5α Reductase

**Figure 5:**
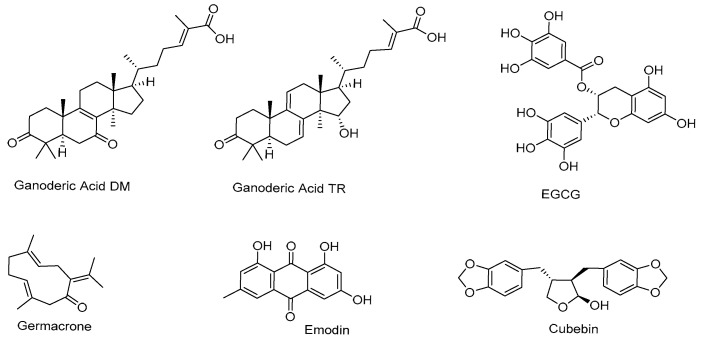
Chemical structures of ganoderic acid DM, ganoderic acid TR, EGCG, germacrone, emodin, and cubebin.

5α-Reductase, the enzyme responsible for converting testosterone into the more potent androgen DHT, has emerged as a critical target in the search for natural product-based therapeutic for prostate cancer. Inhibiting 5α-reductase offers a promising strategy to reduce androgenic stimulation in prostate cancer. A few naturally occurring compounds with diverse chemical structures (Figure 5), including ganoderic acid DM, ganoderic acid TR, (-)-epigallocatechin-3-gallate (EGCG), sesquiterpenes, emodin, and cubebin, have been experimentally shown to inhibit 5α-reductase activity.

Ganoderic acid DM, isolated from the fruiting body of *Ganoderma lucidum*, inhibits 5α-reductase with an IC_50_ value of 10.6 μM and demonstrated 72% inhibitory activity at a concentration of 25 μM, with optimal performance observed at pH 7–8 [23,24]. Ganoderic acid TR (15α-hydroxy-3-oxolanosta-7,9(11),24(E)-trien-26-oic acid), also isolated from *Ganoderma lucidum*, inhibits 5α-reductase in a concentration-dependent manner with a IC_50_ value of 8.5 μM [25]. Structure-activity relationship studies revealed that its carboxylic acid side chain is essential for the activity, as methylation diminishes its inhibitory potency. Although increased concentrations further reduced enzyme activity, complete inhibition was not achieved.

EGCG, the principal polyphenol in green tea, has also been reported to suppress 5α-reductase activity, limiting the conversion of testosterone to DHT [26]. Sesquiterpenes, a class of naturally occurring terpenes (C_15_H_24_), are abundant in plants such as *Curcuma aeruginosa*. Six sesquiterpenes, germacrone, zederone, dehydrocurdione, curcumenol, zedoarondoil, and isocurcumenol, were identified by Suphrom and colleagues as inhibitors of 5α-reductase [27]. Among these, germacrone exhibited the most potent antiandrogenic potency, exceeding that of ethinylestradiol, significantly inhibiting flank gland growth (comparable to finasteride) and reducing the viability of androgen-responsive LNCaP prostate cancer cells. Emodin, an anthraquinone compound, acts as a 5α-reductase inhibitor with an IC_50_ value of 40 μM. Structure–activity relationship studies emphasize the essential role of emodin’s hydroxyl groups in its inhibitory effect [28]. Lastly, natural products from *Piper cubeba*, a medicinal plant native to Indonesia, have also demonstrated 5α-reductase inhibition. The ethanolic extract P9605 and its major constituent cubebin selectively inhibit 5α-reductase type II and attenuate AR signaling, reducing proliferation in androgen-dependent prostate cancer cells [29].

## 5. Natural Products as Androgen Receptor Antagonists

### 5.1. Interacting with LBD

**Figure 6:**
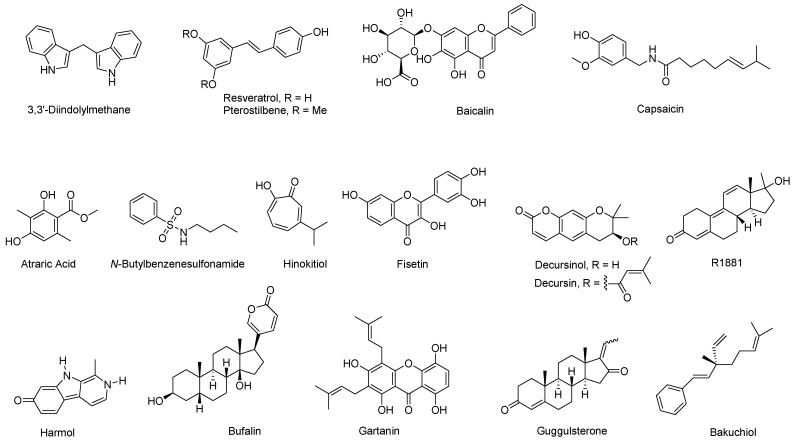
Chemical structures of reseratrol, baicalin, capsaicin, atraric acid, *N*-butylbenzenesulfonamide, hinokitiol, fisetin, decursin, decursinol, harmol, bufalin, gartanin, guggulsterone, and bakuchiol.

Multiple natural compounds exhibit antagonistic effects on AR by targeting its ligand-binding domain (LBD) and disrupting essential receptor functions (Figure 6). Ganoderic acid DM, aside from being an effective 5α-reductase inhibitor, competes with DHT for AR binding in prostate cancer cells [30]. It inhibits the viability and growth of AR-positive LNCaP cells even in the presence of androgens such as DHT and further suppresses osteoclastogenesis [30].

EGCG is a potent AR antagonist acting through multiple pathways. It competitively binds to the AR-LBD (IC_50_ = 0.4 μM), disrupting high-affinity androgen binding and inhibiting AR transcriptional activation [31]. EGCG displaces labeled androgen ligands such as Fluormone AL Red and reduces FRET signals, which confirms its direct competition with androgen ligands. This interaction also inhibits *N*-terminal/*C*-terminal (*N*–*C*) interdomain communication that is indispensable for AR’s transcriptional function.

3,3′-Diindolylmethane (DIM), derived from cruciferous vegetables, is a competitive AR antagonist with an affinity comparable to bicalutamide [32,33]. DIM inhibits DHT binding, reduces AR nuclear translocation, and downregulates AR target genes such as PSA. Clinically, BR-DIM (formulated DIM capsule designed for enhanced bioavailability) treatment in localized prostate cancer patients resulted in reduced nuclear AR and decreased PSA levels [33].

Resveratrol, a polyphenol abundant in grapes and berries, disrupts AR signaling by antagonizing AR via binding to the LBD and inhibiting its transcriptional activity, nuclear translocation, and acetylation [34,35]. It reduces AR expression at transcriptional and protein levels and suppresses androgen-responsive genes, including PSA and hK2. Analogs such as pterostilbene exhibit enhanced potency due to favorable binding interactions with AR-LBD [35].

Baicalein, a flavonoid from *Scutellaria baicalensis*, has been demonstrated to inhibit AR transactivation, disrupt *N*-*C* interactions, and interfere with AR-coactivator binding, leading to AR signaling disruption [36].

Capsaicin, isolated from *Capsicum* species, inhibits AR signaling in prostate cancer [37]. It acts as an AR antagonist by blocking DHT-mediated PSA promoter activation, reducing PSA and AR levels.

Atraric acid, a phenolic compound isolated from *Pygeum africanum*, competitively antagonizes AR by binding to the LBD, inhibiting nuclear translocation, and suppressing androgen-induced AR activity [38]. Structure-activity relationship studies show that its *para*-hydroxy group and hydrophobic substitutions enhance potency.

*N*-butylbenzene-sulfonamide (NBBS), a natural compound also isolated from *Pygeaum africanum*, inhibits androgen-induced AR transactivation and prevents AR nuclear translocation by binding to the LBD [39]. Structure-activity relationship studies reveal that *para*-unsubstituted benzene ring and hydrophobic side chains were essential for the activity.

Hinokitiol, also known as β-thujaplicin, is a monoterpenoid compound isolated from the heartwood of *Cupressaceae* plants. It inhibits AR-positive prostate cancer cell proliferative by competitively displacing radiolabeled androgens and downregulating AR mRNA and protein levels in a dose-dependent manner [40]. Furthermore, hinokitiol markedly decreased both intracellular and secreted PSA levels under androgen-stimulated conditions, as confirmed by Western blot and ELISA analysis.

*Saw palmetto*, a small palm plant rich in fatty acids and phytosterols, has been demonstrated to interfere with AR signaling by reducing DHT- and IL-6-induced PSA expression and nuclear AR accumulation, while selectively blocking IL-6-mediated STAT3 phosphorylation [41].

Cryptotanshinone possesses a chemical structure closely resembling to that of DHT. It selectively inhibits DHT-induced AR transactivation without interfering with other nuclear receptors such as estrogen receptor α (ERα), glucocorticoid receptor (GR), or progesterone receptor (PR) [42]. It inhibited AR-positive prostate cancer cell proliferation (LNCaP and CWR22Rv1) at 0.5 μM, while exerting minimal effects on AR-negative PC-3 cells and non-malignant RWPE-1 prostate epithelial cells. Mechanistically, cryptotanshinone inhibits AR dimerization and coactivator complex formation, particularly by blocking interactions between AR and ARA70 (a coactivator), as well as the *N*-*C* interaction. Although it significantly downregulated AR target genes (e.g., PSA, TMPRSS2, TMEPA1) at the mRNA level, it did not affect AR protein expression or stability [42].

Fisetin, a dietary flavonol found in strawberries, apples, kiwis, and cucumbers, has been shown to inhibit AR signaling in prostate cancer cells by directly targeting AR-LBD region and disrupting the interaction between *N*-*C* terminals [10,43]. FRET-based competitive binding assays confirmed that fisetin competes with endogenous androgens for binding to AR-LBD, reducing the time-resolved FRET signal and indicating displacement of labeled androgens. Importantly, co-treatment with the synthetic androgen R1881 and fisetin further diminished AR *N*-*C* terminal interaction at concentrations of 10 and 20 μmol/L, confirming fisetin’s role as an AR antagonist. In addition to competitive AR binding, fisetin reduces AR targeted gene levels through transcriptional and post-translational mechanisms. Reporter assays showed that fisetin treatment (40 and 60 μmol/L) significantly suppressed AR gene promoter activity, which correlated with decreased AR mRNA levels at these concentrations.

Decursin and decursinol, two pyranocoumarins isolated from *A. gigas*, were identified as competitive AR antagonists [44]. Harmol hydrochloride, a harmala alkaloid isolated from *Fontinalis squamosa*, was established as a selective and competitive AR antagonist by binding to the LBD [45]. Its antagonism is more potent than bicalutamide, an FDA approved first-generation nonsteroidal AR antagonist for the treatment of metastatic prostate cancer [46]. Bufalin, a natural cardiotonic from *Bufo gargarizans*, was established as an AR antagonist, but its clinical use is limited by severe cardiac toxicity due to Na^+^/K^+^-ATPase inhibition. Structural modifications, such as Δ^8,14^-anhydrobufalin, bufadienolactam, and secobufalinamide, significantly improved AR binding affinity and reduced cardiac side effects, highlighting their potential as safer anti-PCa agents [47,48]. Gartanin, a xanthone isolated from *Garcinia mangostana*, is an AR antagonist with comparable binding affinity (10.8 μM) to the first generation nonsteroidal AR antagonist flutamide (10.3 μM) in fluorescence polarization competitive binding assays [49]. Cell-based FRET assays confirmed its AR antagonist activity with an EC_50_ of approximately 8.5 μM. In silico molecular modeling further supported these findings, demonstrating gartanin’s similar binding mode to testosterone within the LBD. Guggulsterone, the primary component of myrrh resin from Ayurvedic medicinal plants *Commiphora mukul* and *Commiphora wightii*, exists as two stereoisomers, *E*-guggulsterone and *Z*-guggulsterone. Both stereoisomers exhibit significant AR antagonism, with IC_50_ values of 660 nM for the *E*-isomer and 220 nM for the *Z*-isomer in cell-based transfection assays [50,51]. Additionally, they effectively antagonize AR signaling stimulated by R1881 in HEK293 and C2C12 cell lines. Bakuchio, isolated from *Psoralea corylifolia*, binds to AR with an affinity similar to that of flutamide [52].

### 5.2. Interacting with the N-Terminal Domain (NTD)

**Figure 7:**
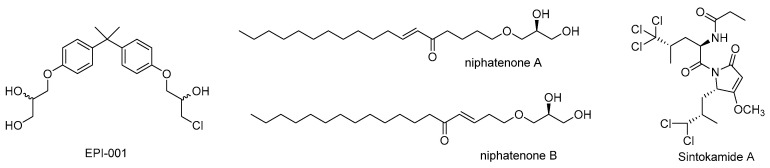
Chemical structures of EPI-001, niphatenones A & B, and sintokamide A.

Unlike the structured LBD, the AR *N*-terminal domain (NTD) is intrinsically disordered yet essential for transcriptional activation via the activation function-1 (AF-1) region. Targeting the NTD can suppress both full-length AR and constitutively active splice variants that lack the LBD, offering a strategy to overcome resistance to LBD-targeting drugs. A few natural products (Figure 7), including EPI-001, niphatenones A& B, and sintokamide A, have been established the first exemplified compounds that can interact with AR NTD.

EPI-001, derived from a marine sponge, is the first small molecule reported to covalently bind to the AF-1 region of the AR NTD. This interaction disrupts critical protein–protein interactions required for AR transcriptional activity, making EPI-001 effective in both hormone-sensitive and castration-resistant prostate cancer cells, including those expressing AR splice variants lacking the LBD [53]. LBD-binding AR antagonists such as bicalutamide and enzalutamide competitively bind the LBD and are susceptible to resistance with elevated androgen levels. However, EPI-001 retains inhibitory effects even at high androgen concentrations (e.g., 50 nM R1881), as shown in PSA-luciferase assays in LNCaP cells [53]. EPI-001 selectively interferes with AR–CBP interactions without disrupting AR–SRC1/2/3 binding, and maintains suppression of AR activity despite overexpression of coactivators [54]. Structural studies using solution NMR [55] demonstrated that EPI-001 interacts reversibly and covalently with the partially folded Tau-5 region within AF-1, causing specific chemical shift changes and modulating AR transactivation. In xenograft models, EPI-001 downregulates AR target genes and inhibits tumor growth [56].

Niphatenones A and B are glycerol ether lipids isolated from the marine sponge *Niphates digitalis* that inhibit both full-length AR and AR NTD transcriptional activity and disrupt the *N*-/*C*-terminal interaction. In a PSA-luciferase assay in LNCaP cells treated with 1 μM R1881 (synthetic androgen) and 7 μM niphatenone enantiomers, niphatenone B inhibited AR transcriptional activity by approximately 50%, while niphatenone A reduced activity by 25% [57]. The (*R*)-niphatenone B shows the highest potency, followed by (*R*)-niphatenone A, (*S*)-niphatenone B, and (*S*)-niphatenone A, suggesting the importance of the glycerol C-2′ chiral center for AR antagonism [57]. In 2014, Banuelos et al. further elucidated niphatenone B’s mechanisms, demonstrating inhibition of AR NTD transactivation, *N*-/*C*-terminal interaction, and ligand binding at higher concentrations, without altering AR protein levels or localization [58,59,60]. Both (*S*)- and (*R*)-niphatenone B inhibited transcriptional activity of the AR splice variant ARV-567 by 40% at 1 μM, but showed no effect in AR-null PC-3 cells.

Sintokamide A (SINT1), a small peptide isolated from the marine sponge *Dysidea species*, has been shown to selectively suppress AR activity in LNCaP prostate cancer cells by inhibiting transactivation of the AR NTD, specifically targeting the AF-1 region. This antagonism is selective without affecting other steroid receptors such as PR or GR [61]. Additionally, SINT1 did not bind to the AR LBD and did not alter AR nuclear localization.

## 6. Natural Products Degrading AR

**Figure 8:**
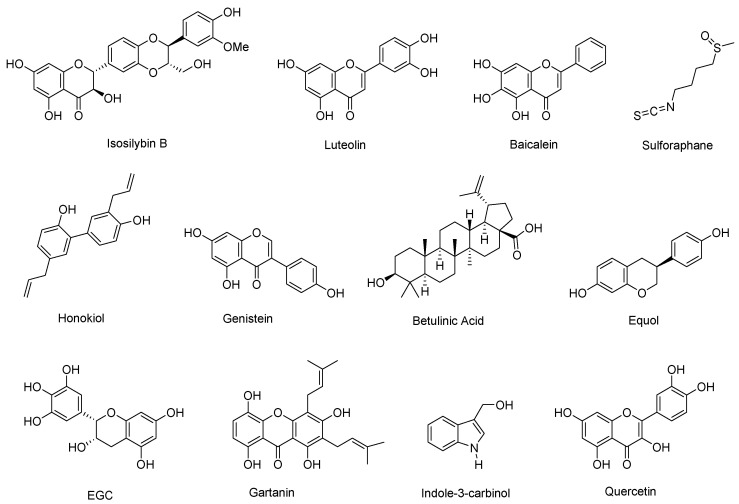
Chemical structures of isosilybin B, luteolin, baicalein, sulforaphane, honokiol, genistein, betulinic acid, equol, EGC, gartanin, indole-3-carbinol, quercetin.

AR degradation emerges as a promising therapeutic strategy to combat aggressive prostate cancer, especially to overcome drug-resistance [62]. Numerous natural products (Figure 8) have been identified to destabilize AR protein, promote its ubiquitination, and facilitate proteasome-mediated degradation, leading to the disruption of downstream transcriptional programs such as PSA expression. These compounds act through diverse mechanisms, including interference with AR-Hsp90 chaperone interactions, modulation of histone deacetylase activity, inhibition of deubiquitinases, and suppression of androgen biosynthesis. The following section summarizes key natural products (Figure 7) that have been shown to promote AR degradation in prostate cancer cells.

Isosilybin B promotes AR degradation in prostate cancer cells by disrupting the PI3K-Akt-Mdm2 signaling pathway [63]. In this pathway, Akt mediates AR phosphorylation, while Mdm2 facilitates its ubiquitination, collectively driving AR degradation [64]. Specifically, Akt phosphorylates AR at Ser-210 and Ser-790, which suppresses AR transactivation and inhibits AR-induced apoptosis. Isosilybin B has been demonstrated to enhance Akt phosphorylation at Ser-473 and Thr-308 in LNCaP cells, as well as Mdm2 phosphorylation at Ser-166 in both LNCaP and 22Rv1 cell lines [63]. These phosphorylation events are linked to the activation of Mdm2’s ubiquitin ligase activity, ultimately promoting AR degradation.

Curcumin consistently reduces AR levels and activity in prostate cancer cells. In AR-positive LNCaP cells, treatment with curcumin (10–20 µM) markedly downregulates both AR protein and mRNA. This process in turn decreases AR binding to androgen response elements (AREs) and suppresses the expression of AR target genes such as PSA [65,66]. For example, curcumin inhibits R1881- and interleukin-6-induced PSA expression in LNCaP cells by lowering AR expression and reducing AR recruitment to the PSA gene promoter. In mice bearing LNCaP xenograft tumors, curcumin administration significantly slowed tumor growth and was shown to suppress tumor AR expression and PSA production in vivo [67].

Luteolin, a natural flavonoid, exhibits potent antiproliferative effects on prostate cancer cells, particularly AR-positive LNCaP cell line. Its activity is dose- and time-dependent, leading to growth inhibition, and suppression of AR signaling and PSA expression. Mechanistically, luteolin destabilizes AR protein by disrupting the AR-Hsp90 interaction, promoting AR ubiquitination and facilitating proteasome-mediated AR degradation [68].

Baicalein inhibits both androgen-sensitive LNCaP and androgen-insensitive JCA-1 prostate cancer cell lines, displaying greater potency than its glycoside precursor, baicalin. In early studies, baicalein was shown to suppress AR expression in both nuclear and cytoplasmic compartments [69]. These findings were later reinforced by Bonham et al., who reported a significant reduction in AR and PSA protein levels, accompanied by significant disruption of cell cycle progression [70].

Sulforaphane, a sulfur-rich compound abundant in cruciferous vegetables such as broccoli and cabbage, has shown potential in both the prevention and treatment of prostate cancer through modulating AR transcriptional signaling. Its activity is mediated in part by inhibition of histone deacetylases, particularly HDAC6, which enhances Hsp90 acetylation, disrupts the Hsp90–AR complex, and promotes AR degradation. Sulforaphane has been revealed to increase acetylated Hsp90 and α-tubulin levels while reducing AR–Hsp90 interactions in LNCaP and VCaP cells, resulting in decreased AR stability and downregulated expression of downstream targets such as PSA and TMPRSS2-ERG in a dose-dependent manner [71]. Complementary findings by Kim et al. further demonstrated that sulforaphane suppresses AR mRNA expression, reduces AR promoter activity, and inhibits androgen-stimulated AR nuclear translocation in LNCaP and C4-2 cells [72]. Consistently, Khurana et al. reported a time- and dose-dependent decline in AR protein levels, confirming sulforaphane’s ability to promote AR degradation and inhibit AR gene expression [73].

Honokiol, a lignan isolated from various *Magnolia* species such as *M. grandiflora*, *M. officinalis*, and *M. dealbata*, acts on the AR transcriptional signaling pathway by inhibiting AR nuclear translocation (both in the presence and absence of androgen stimulation), suppressing PSA secretion, and reducing AR protein expression [74]. In prostate cancer cell lines including LNCaP, C4-2, and TRAMP-C1, honokiol significantly decreased AR levels in a dose-dependent manner, with significant effects observed after 48 h of treatment [74]. Honokiol analogs, including honokiol dichloroacetate, honokiol epoxide, and biseugenol, were also evaluated, with honokiol dichloroacetate showing the strongest AR suppression and biseugenol the weakest. Importantly, p53 knockdown had little effect on honokiol-induced AR inhibition, suggesting a p53-independent mechanism.

Genistein, an isoflavone originally isolated from dyer’s broom (*Genista tinctorial*), suppresses AR signaling primarily by promoting AR degradation. Its mechanism involves proteasome-mediated degradation of AR through disruption of the HDAC6-Hsp90 chaperone complex [75]. Under normal conditions, Hsp90 stabilizes AR, and this function is regulated by its acetylation status. Genistein enhances Hsp90 acetylation by reducing both the levels and activity of HDAC6, a deacetylase and Hsp90 co-chaperone, thereby impairing Hsp90’s chaperone function. As a result, AR undergoes increased ubiquitination and subsequent proteasomal degradation [75]. This mechanism has been validated in AR-positive LNCaP prostate cancer cells, where genistein induced dose-dependent reduction in AR protein and transcriptional activity. At 25 µM, genistein effectively downregulated AR expression, while higher concentrations promoted accumulation of polyubiquitinated AR forms [75]. Interestingly, genistein’s effects on AR degradation were also linked to estrogen receptor- β (ER-β), suggesting that its inhibitory activity both ubiquitination-mediated proteasomal degradation and ER-β-dependent signaling [76].

Betulinic acid, a pentacyclic triterpenoid isolated from species such as white birch (*Betula pubescens*) and the ber tree (*Ziziphus mauritiana*), exerts anti-prostate cancer effects primarily through AR degradation. Reiner et al. demonstrated that betulinic acid inhibits deubiquitinase activity, leading to accumulation of polyubiquitinated proteins and enhanced proteasomal degradation of AR [77]. This mechanism was correlated with reduced AR levels in tumor tissues and suppressed tumor growth in TRAMP mice. Extending these findings, de las Pozas et al. demonstrated that betulinic acid also downregulates AR mRNA expression by inhibiting a histone 2A-specific DUB, which increases ubiquitinated histone 2A, a transcriptional repressor [78].

Emodin promotes AR degradation by increasing ubiquitination, primarily through disrupting AR-Hsp90 interactions and enhancing the recruitment of AR to the E3 ubiquitin ligase MDM2. Importantly, emodin does not alter AR mRNA expression, suggesting that its regulatory effect occurs post-translationally by destabilizing AR protein [79].

Fisetin promotes AR protein degradation by antagonizing androgen-mediated AR stabilization. In cells exposed to DHT, which normally enhances AR protein stability, co-treatment with 40 μM fisetin significantly reduced AR levels. In cycloheximide-based pulse-chase experiments, fisetin accelerated AR degradation, decreasing AR half-life from 18 h to 4.5 h. These results indicate that fisetin destabilizes AR protein through interference with ligand-induced stabilization [10].

Berberine, a natural isoquinoline alkaloid derived from *Berberis* and *Coptis* species also suppresses AR signaling. It reduces AR transactivation, and impairs AR nuclear translocation without significantly affecting AR mRNA levels [80]. In LNCaP, LAPC-4, C4-2B, and PC-3 cells, berberine decreased cell viability in a dose- and time-dependent manner (IC_50_: 3.4–70.2 µM) and suppressed both ligand-dependent and ligand-independent AR activity, including in AR-variant-expressing 22Rv1 cells. Mechanistically, berberine reduced the expression of AR-regulated genes such as PSA and DHCR24. Mechanistically, it induced AR protein degradation, likely via the proteasome pathway, and disrupted AR-Hsp90 complex formation, thereby inhibiting nuclear translocation [80].

Equol, a metabolite of soy isoflavones, promotes AR degradation by modulating S-phase kinase-associated protein 2 (Skp2), which regulates AR expression through ubiquitin-mediated proteolysis. Treatment with equol enhanced AR-Skp2 interactions, reduced AR and Skp2 protein levels, and increased accumulation of the CDK inhibitor p27 by decreasing its ubiquitination. These effects suppressed AR-positive prostate cancer cell growth and induced apoptosis, with Skp2 downregulation occurring in a cell line-dependent manner [81].

Gartanin also enhances AR degradation, resulting in reduced AR protein levels and suppression of PSA expression, thus disrupting AR/PSA signaling. In parallel, gartanin modulates endoplasmic reticulum stress proteins and chaperones, which may contribute to AR destabilization [49].

Other natural compounds demonstrate AR-suppressive effects through related mechanisms. Epigallocatechin (EGC) and EGCG reduce histone acetyltransferase activity [82]. Indole-3-carbinol, originated from *Brassica* vegetables, downregulates AR expression at both the mRNA and protein levels. In LNCaP cells, indole-3-carbinol inhibits AR promoter activity and reduces expression of the AR-regulated gene PSA [83]. Similarly, quercetin suppresses AR and PSA expression in LNCaP cells [84].

## 7. Natural Products Suppressing AR Signaling via Other Pathways

**Figure 9:**
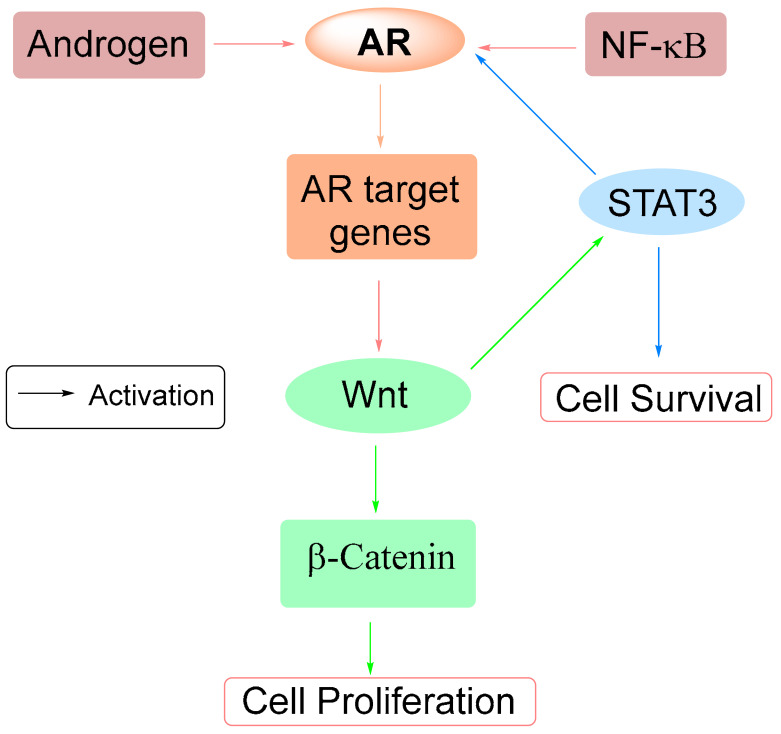
Schematic representation of crosstalk between AR signaling pathway and NF-κB, Wnt/β-catenin, and STAT3.

In addition to suppressing androgen biosynthesis, acting as AR antagonists, and promoting AR degradation, several natural products suppress AR signaling through alternative mechanisms. These include interference with nuclear translocation, modulation of AR splice variants, disruption of co-regulator interactions, and regulation of cross-talk pathways such as NF-κB, Wnt/β-catenin, STAT3, and receptor tyrosine kinases. Others act through epigenetic or microRNA-mediated regulation, further broadening their impact on AR signaling. These interactions are illustrated in Figure 9, which summarizes the crosstalk between AR signaling and NF-κB, Wnt/β-catenin, and STAT3 in prostate cancer. The representative compounds that exemplify the diversity of these indirect mechanisms are presented below.

EGCG suppresses AR signaling by blocking nuclear translocation and reducing AR protein levels in prostate cancer xenografts through sequestration of AR in the cytoplasm. Mechanistically, EGCG modulates AR-regulated microRNAs, downregulating oncogenic miRNA-21 while upregulating tumor-suppressive miRNA-330, which modulates the AKT/P-AKT/caspase-3/MMP-2/MMP-9 signaling axis [31,85]. EGCG also disrupts AR signaling through inhibition of NF-κB by preventing RelA acetylation [86] and by suppressing histone acetyltransferases, which reduces AR acetylation and nuclear localization [82].

Curcumin disrupts the interaction between AR and *β*-catenin in LNCaP prostate xenograft model, which attenuates downstream TCF target gene expression [87]. Curcumin also reduced the expression or activity of receptor tyrosine kinases (EGFR/HER2) that can crosstalk with AR signaling in both LNCaP and C4-2B cells [88].

Luteolin further interferes with AR signaling by targeting the splice variant AR-V7, a driver of CRPC. It induces miR-8080, which directly binds the AR-V7 mRNA 3′-UTR, resulting in reduced AR-7 expression and inhibition of AR-V7-driven signaling [89].

In addition to acting as an AR antagonist, capsaicin displays the other dual effects on AR regulation. At low concentrations (≤20 μM), it may stimulate AR expression via PI3K and MAPK signaling [90]. Capsaicin also restores miR-449a expression, which downregulates AR and PSA, enhancing cell sensitivity to capsaicin treatment [91].

**Figure 10:**
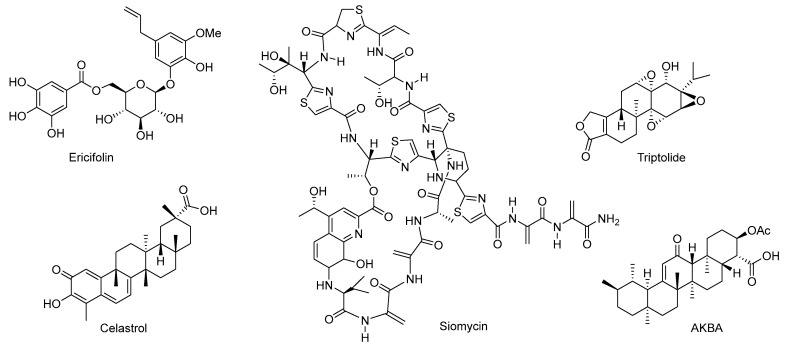
Chemical structures of ericifolin, siomycin A, triptolide, celastrol, and acetyl-11-keto-beta-boswellic acid (AKBA).

Ericifolin (Figure 10), a purified bioactive compound from aqueous allspice extract (AAE), suppresses AR signaling by reducing AR promoter activity and mRNA stability [92]. Treatment with AAE significantly reduces AR protein levels in AR-positive cell lines such as LNCaP and LAPC-4, without substantially affecting AR protein stability. This decrease was not due to proteasomal degradation but rather transcriptional inhibition, as confirmed by experiments involving the proteasome inhibitor MG132. Additionally, AAE treatment dose-dependently decreased AR mRNA expression and repressed AR promoter activity at concentrations ≤10 μg/mL, leading to reduced PSA secretion by approximately 43% at 100 μg/mL and 53% at 150 μg/mL. Given ericifolin exhibited similar regulatory effects on AR mRNA expression, apoptosis, and cell cycle regulation at concentrations comparable to AAE, it suggests ericifolin possesses potential capability in suppressing AR signaling pathway and chemoprotective properties analogous to AAE [92].

Siomycin A (Figure 10), a thiazole antibiotic, selectively inhibit AR activity in neuronal but not muscle cell lines. Identified through high-throughput screening, siomycin A reduced DHT-induced AR conformational changes and AR transcriptional activity in neuronal GT1-7 cells but showed minimal effects in C2C12 muscle cells [93]. Its neuron-specific AR inhibition was attributed to suppression of FOXM1, a transcription factor that modulates AR signaling. Thiostrepton, another thiazole antibiotic, acts through a similar FOXM1-dependent mechanism but demonstrates greater potency, suppressing both wild-type and mutant AR in neuronal models and disrupting disrupts the interaction between *β*-catenin and AR [93].

Triptolide (Figure 10), a diterpene triepoxide isolated from *Tripterygium wilfordii* Hook F, inhibits AR signaling through multiple mechanisms. It downregulates full-length AR and splice variants, suppresses AR transcriptional activity, and reduces expression of AR targets such as PSA and NKX3.1 [94]. Mechanistically, triptolide inhibited Sp1 expression and nuclear translocation, induces AR degradation via calpain activation, and blocks phosphorylation of AR and AR-V7 at Ser515 by inhibiting XPB/CDK7, impairing chromatin binding and recruitment of transcriptional machinery [95,96].

Celastrol (Figure 10), another compound isolated from *Tripterygium wilfordii*, acts as a natural proteasome inhibitor. It has been demonstrated to modulate apoptotic proteins and inhibit the constitutive activation of NF-κB, a pro-survival factor inversely correlated with AR status. Elevated NF-κB activity, often associated with high proteasome function, contributes to disease progression and therapeutic resistance. By acting as a proteasome inhibitor, celastrol disrupts NF-κB signaling via stabilization of cytosolic IκBα and inhibition of RelA/p65 nuclear translocation. In vitro studies using prostate cancer cell lines (LNCaP, PC-3, DU-145, and CL1) revealed that celastrol significantly reduced cell viability in a dose-dependent manner, with IC_50_ values ranging from 0.64 to 1.54 μM. Celastrol treatment also effectively suppressed NF-κB nuclear translocation, similar to the known proteasome inhibitor MG132. These findings highlight celastrol’s potential as a therapeutic agent targeting proteasome-dependent AR signaling pathways in advanced prostate cancer [97].

Decursin inhibits AR signaling indirectly by disrupting the Wnt/β-catenin signaling, downregulating cyclin D1 and c-myc, and suppressing androgen-independent PC3 prostate cancer cell proliferation [98,99]. Similarly, saw palmetto blocks IL-6-induced STAT3 phosphorylation by 60% without affecting total STAT3 levels, which interferes with AR-associated STAT3 signaling [41].

Acetyl-11-keto-*β*-boswellic acid (AKBA) (Figure 10), a pentacyclic triterpenoid derived from *Boswellia carterii*, reduces DHT-induced AR expression at both mRNA and protein levels and disrupts AR transcriptional regulation by interfering with Sp1 binding to the AR promoter [100,101]. In LNCaP cells, AKBA decreased DHT-stimulated AR protein and reduced AR mRNA expression by 3.4-fold. Luciferase reporter assays confirmed that AKBA suppresses DHT-induced activation of the AR promoter and PSA promoter. Furthermore, in both LNCaP and PC3 cells transfected with AR, AKBA inhibited androgen-responsive element (ARE)-driven luciferase activity, indicating a reduction in AR transcriptional activity [101]. Additional experiments showed that while AKBA diminished Sp1-mediated activation of the AR short promoter, it did not alter Sp1 protein expression, suggesting selective interference with Sp1 binding rather than expression [101].

Equol inhibits AR signaling indirectly by binding DHT with high affinity, sequestering it from AR [102]. This inhibition prevents androgen-driven transcription and prostate cell growth. Bioavailable formulations such as BR-DIM and B-DIM enhance these effects by suppressing AR transcription, inhibiting Akt and NF-κB signaling, and inducing apoptosis in AR-positive LNCaP and C4-2B cells [7].

Cryptotanshinone suppresses AR signaling by targeting lysine-specific demethylase 1 (LSD1), an AR coactivator. By disrupting AR–LSD1 complex formation at AR-responsive promoters and increasing repressive H3K9 methylation, cryptotanshinone attenuates AR transcriptional activity [103].

## 8. Promising Scaffolds and Translational Potential

To provide a consolidated overview of the compounds discussed, the molecular targets and mechanisms of action of natural products interacting with AR signaling pathway are summarized in Table 1.

Although numerous natural products modulate AR signaling, a subset stands out as potential scaffolds for therapeutic development. Compounds such as cryptotanshinone, EGCG, DIM/BR-DIM, fisetin, gartanin, guggulsterone, sulforaphane, berberine, curcumin, and luteolin have demonstrated selective AR-directed activity, inhibition of AR splice variants, or in vivo antitumor efficacy. Importantly, agents targeting the AR NTD, such as EPI-001 and sintokamide A, retain activity against constitutively active AR variants, suggesting their translational relevance for CRPC. Collectively, these compounds represent priority scaffolds for further optimization.

**Figure 11:**
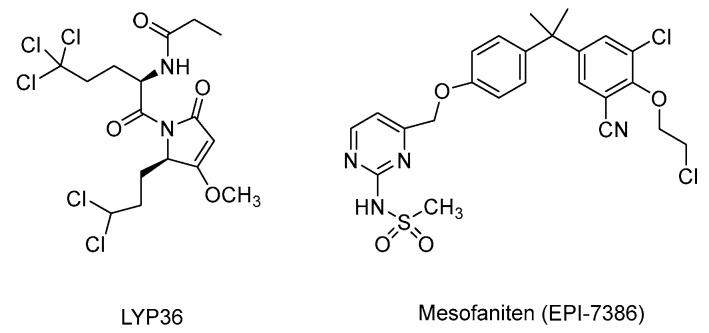
Chemical structures of mesofaniten and LYP36.

In addition to their intrinsic activity, several natural products have served as templates for analog and formulation development. Resveratrol led to the design of pterostilbene (Figure 6), a demethylated analog with greater metabolic stability and potency. DIM has been formulated as BR-DIM, a preparation with improved bioavailability and documented AR-modulating effects. Optimization of sintokamide through structure-activity studies produced LPY36 (Figure 11), which exhibits greater selectivity, higher potency, improved synthetic accessibility, and enhanced stability compared to the parent compound [104]. The most clinically advanced example is the development of masofaniten (EPI-7386) (Figure 11) from EPI-1 [105], which progressed into multiple Phase II clinical as monotherapy (NCT04421222) or in combination with enzalutamide (NCT05075577), abiraterone acetate plus prednisone or apalutamide (NCT05295927), or enzalutamide with androgen deprivation therapy (NCT06312670). Although these trials were discontinued by ESSA Pharma due to a low likelihood of achieving the primary endpoint (≥90% PSA response rate) in a combination therapy, they highlight the potential and challenges of translating natural product scaffolds into clinically effect AR-targeted agents. Together, these examples illustrate the dual role of natural products as direct modulators and as productive scaffolds for rational drug design.

The therapeutic implications of these scaffolds are significant. Compounds such as cryptotanshionon, DIM/BR-DIM, and fisetin act as competitive antagonists with favorable selectivity; sulforaphane, genistein, and betulinic acid promote AR degradation through proteostasis pathways; and curcumin, berberine, and luteolin not only suppress AR signaling but also modulate compensatory mechanisms, including NF-κB, Wnt/*β*-catenin, or STAT3. This multifaceted activity may be advantageous in advanced or treatment-resistant disease, where adaptive signaling and AR variants undermine the efficacy of conventional AR-targeting therapies. Taken together, these observations underscore that natural products are not merely a diverse catalog of AR modulators but represent a strategic source of selective, translatable, and optimizable scaffolds. By integrating mechanistic insight with analog development, these agents provide a rational foundation for drug discovery efforts aimed at overcoming therapeutic resistance and expanding treatment options in prostate cancer.

## 9. Conclusions and Future Directions

Collectively, natural products have emerged as versatile modulators of AR signaling, acting through diverse mechanisms that include inhibition of testosterone biosynthesis, suppression of 5α-reductase, direct AR antagonism, induction of AR degradation, and disruption of AR signaling through transcriptional, epigenetic, and co-regulatory pathways. These multifaceted actions underscore their potential to target both ligand-dependent and castration-resistant prostate cancer, including disease driven by AR splice variants. While sufficient in vitro evidence strongly supports their therapeutic promise, challenges such as poor bioavailability, complex multi-target effects, and limited in vivo and clinical validation remain barriers to translation. Future work focused on structural optimization, novel delivery systems, and rigorous clinical evaluation will be critical to harness natural products as effective and sustainable strategies for prostate cancer therapy. Future studies should also investigate the potential of combining natural products with new-generation AR antagonists such as enzalutamide, apalutamide, and darolutamide, as these strategies may further enhance therapeutic efficacy and overcome resistance mechanisms.

## Figures and Tables

**Table 1:** Natural products, their primary molecular targets, and mechanisms of action on the AR signaling pathway.

Natural Product	Primary Molecular Target	Mechanism of Action on AR Signaling	References
Glycyrrhetinic acid	17,20-lyase, 17β-HSD	Inhibits steroidogenic enzymes Suppresses testosterone biosynthesis	[12,13]
Paeoniflorin	Aromatase	Promotes conversion of testosterone to estrogen	[14]
Carvone	Endocrine modulation	Reduces testosterone levels in animal and clinical studies	[15,16]
Curcumin	StAR, CYP11A1, HSD3B2, AKR1C2	Inhibits steroidogenesis and promotes androgen inactivation	[17,65,66,67]
Apigenin	HSD3B, SYP17A1, HSD17B3	Inhibits key steroidogenic enzymes	[18]
Cryptotanshinone	ERK/c-Fos/CYP17, AR-LBD, LSD1	Suppresses steroidogenesis Selective AR antagonist Disrupts AR-LSD1	[19,42,103]
Berberine	AKR1C3, AR-Hsp90 complex	Inhibits androgen biosynthesis Destabilizes AR	[20,21,22,80]
Ganoderic Acids (DM/TR)	5α-reductase AR-LBD	Inhibit DHT synthesis Compete with AR binding	[23,24,25,30]
EGCG	AR-LBD, NF-κB	Competes with AR ligan Inhibits AR nuclear translocation Modulates miRNAs	[26,31,82,85,86]
DIM/BR-DIM	AR-LBD	Competitive antagonist Inhibits AR nuclear translocation	[32,33]
Resveratrol/Pterostilbene	AR-LBD	Antagonist, Inhibits AR transcriptional activity	[34,35]
Fisetin	AR-LBD, AR stability	Competes with androgens Promotes AR degradation	[10,43]
Sulforaphane	HDAC6-Hsp90-AR complex	Destabilizes AR, Enhances ubiquitination	[71,72,73]
Luteolin	AR-V7, miR-8080	Suppresses AR-V7 via miRNA regulation	[89]
EPI-001	AR-NTD (AF-1 domain)	Covalently binds to NTD Disrupts AR transcription	[53,54,55,56]
Sintokamide A/LYP36	AR-NTD	Selective NTD antagonist	[61,104]
Masofaniten (EPI-7386)	AR-NTD	Optimized derivative of EPI-001 Progressed to Phase II clinical trials.	NCT04421222 NCT05075577

## Data Availability

No new data were generated in the preparation of this review.

## Abbreviations

The following abbreviations are used in this manuscript:

AF-1	Activation function-1
AKBA	Acetyl-11-keto-*β*-boswellic
AR	Androgen receptor
DHT	Dihydrotestosterone
DIM	3,3′-Diindolylmethane
DUB	Deubiquitinase
EGC	Epigallocatechin
EGCG	Epigallocatechin-3-gallate
GA	Glycyrrhetinic acid
HSP	Heat shock protein
LBD	Ligand binding domain
NBBS	*N*-butylbenzene-sulfonamide
NTD	*N*-terminal domain
PSA	Prostate specific antigen
SAR	Structure-Activity Relationship
SINTI	Sintokamide A
TMPRSS2	Transmembrane protease serine 2
